# Alcohol consumption and risk of gastric cancer: a cohort study of men in Kaunas, Lithuania, with up to 30 years follow-up

**DOI:** 10.1186/1471-2407-12-475

**Published:** 2012-10-15

**Authors:** Ruta Everatt, Abdonas Tamosiunas, Irena Kuzmickiene, Dalia Virviciute, Ricardas Radisauskas, Regina Reklaitiene, Egle Milinaviciene

**Affiliations:** 1 Group of Epidemiology, Institute of Oncology, Vilnius University, Baublio 3B, LT-08406, Vilnius, Lithuania; 2 Laboratory of Population Studies, Institute of Cardiology, Medical Academy, Lithuanian University of Health Sciences, Sukileliu 17, LT-50009, Kaunas, Lithuania

**Keywords:** Alcohol, Alcoholic beverage, Gastric cancer, Cohort studies, Risk factors

## Abstract

**Background:**

Gastric cancer is the second most common cause of death from cancer in the world. Epidemiological findings on alcohol use in relation to gastric cancer remain controversial. The aim of this study was to examine the effect of alcohol consumption on the risk of gastric cancer.

**Methods:**

The association between alcohol intake and the risk of gastric cancer was examined in a population-based cohort of 7,150 men in Kaunas, Lithuania, who were enrolled during 1972–1974 or 1976–1980. After up to 30 years of follow-up, 185 gastric cancer cases were identified. Multivariate Cox proportional hazards models were used to estimate hazard ratios (HR) and corresponding 95% confidence intervals (95% CI). The attained age was used as a time-scale.

**Results:**

After adjustment for smoking, education level and body mass index, the HR of gastric cancer was 2.00 (95% CI: 1.04–3.82) for the highest alcohol consumption frequency (2–7 times per week) compared with occasional drinking (a few times per year) and 1.90 (95% CI: 1.13–3.18) for ≥100.0 g ethanol/week versus 0.1–9.9 g ethanol/week. A stronger effect of alcohol consumption on gastric cancer risk was observed during the second half of the study (1993–2008). In the analysis of gastric cancer risk by alcoholic beverage type, all beverages were included simultaneously in the model. The multivariate HR for men who consumed ≥0.5 litre of wine per occasion (compared with those who consumed <0.5 litre) was 2.95 (95% CI: 1.30–6.68). Higher consumption of beer or vodka was not statistically significantly associated with gastric cancer risk. After adjustment for smoking, education level, body mass index and ethanol, we found no excess risk of gastric cancer in association with total acetaldehyde intake.

**Conclusions:**

This study supports a link between alcohol consumption (primarily from ethanol) and the development of gastric cancer in the Lithuanian population. Although an association with heavy wine consumption was observed, the effect of exposure to acetaldehyde on the development of gastric cancer in this cohort was not confirmed. Further research is needed to provide a more detailed evaluation of alcohol drinking and gastric cancer risk.

## Background

Despite the declining incidence and mortality rates, gastric cancer represents the fourth most common incident cancer and the second most common cause of death from cancer in the world [1]. There were 988,602 new cases and 737,419 deaths in 2010 worldwide [1]. The considerable geographic variation in incidence and mortality rates indicates that environmental and lifestyle factors play an important role in the etiology of gastric cancer. Infection with Helicobacter pylori and smoking are established risk factors; however, alcohol consumption, diet and genetic factors may also play a role in gastric carcinogenesis [2].

Epidemiological studies have reported controversial findings on the association between alcohol consumption and the risk of gastric cancer. In 2007, the International Agency for Research on Cancer classified alcohol consumption as a group 1 human carcinogen, related to cancers of the oral cavity, pharynx, larynx, oesophagus, liver, colorectum, and female breast [3,4]. It was concluded that epidemiological evidence concerning the association between alcohol drinking and gastric cancer is inconclusive. Several published case–control and cohort studies on alcohol and gastric cancer have found no significant association [5-13]. In contrast, other studies reported an increased risk of gastric cancer associated with alcohol consumption [14-19]. An extensive meta-analysis of data published until June 2010 concluded that moderate alcohol consumption is probably not related to gastric cancer, but there was a positive association with heavy alcohol drinking [20]. Risk was higher for gastric noncardia than for gastric cardia adenocarcinoma. In addition, significant heterogeneity in relative risks for heavy alcohol drinking by geographic area was observed, with higher risk among non-Asian studies. However, there are still issues that need to be resolved. The beverage-specific effects were not investigated in the meta-analysis, therefore, it remains unclear whether there is relation between type of alcoholic beverage and gastric cancer. There is a major knowledge gap regarding information about the potential impact of acetaldehyde on cancer risk [21]. Furthermore, little information is available on the long-term effects of alcohol consumption on gastric cancer risk. Whereas there is a high number of epidemiological studies on alcohol drinking in relation to gastric cancer, prospective epidemiological studies in populations with a high risk are lacking.

In Lithuania, gastric cancer incidence and mortality rates among men and women are higher than in most European countries, with incidence rates of 33.8 per 100,000 in men and 14.4 for women in Lithuania, and 16.7 for men and 7.8 for women in 27 countries of the European Union (EU-27) [22]. Similarly, mortality rates are higher in Lithuania (29.9 in men and 10.2 in women) than in EU-27 (12.0 in men and 5.6 in women). Clarifying the role of alcohol consumption in the etiology of gastric cancer is important, because the consumption of alcohol is widespread. A high overall amount of alcohol consumption and a high level of binge drinking as compared to other European countries have been reported in Lithuania: 39% of men drank ≥60 g of pure alcohol on a single occasion at least once per month in 2010 [23,24]. The aim of our study was to evaluate the association between the alcohol consumption (frequency, ethanol intake in grams per week and acetaldehyde intake in milligrams per week) and the risk of gastric cancer in a 30 year population-based cohort study of 7,150 men in Lithuania. We also investigated the impact of the type of alcoholic beverage. In this article we also report findings from a second half of the follow-up period (1993–2008).

## Methods

### Study population

Two cohorts - Kaunas Rotterdam Intervention Study (KRIS) and Multifactorial Ischemic Heart Disease Prevention Study (MIHDPS) - are included. KRIS is a WHO-coordinated prospective cohort study of a random sample of 2,447 men aged 45–59, living in the city of Kaunas (Lithuania), who took part in a cardiovascular screening programme in 1972–1974 (69% of eligible participants). The MIHDPS was carried out in 1977–1980 among 5,933 Kaunas men, aged 40–59, representing 70% of eligible participants. These are prospective population-based studies designed to investigate risk factors for cardiovascular diseases and other health-related outcomes among urban population of middle-aged men from Kaunas. The city of Kaunas is located in central Lithuania, which has about 336,000 inhabitants, while the total population of the Lithuania is 3.2 million.

Both studies were based on voluntary, informed participation. Participants gave no written informed consent prior to the baseline examination as this was not required in the former Soviet Union. The current study, including the use of data from the 1970s without written informed patients’ consent, was approved by the Regional Biomedical Research Ethical Committee in 2011 (No. 158200-02-280-65).

### Exposure assessment

All participants underwent physical examination; information on alcohol consumption and potential confounders, including demographic factors and smoking, was obtained via interview. The usual frequency of intake and usual dose for each type of drink (beer, wine, vodka) were recorded using a structured questionnaire [25-27]. Participants were asked how frequently they consumed alcohol, the answer choices were: several times per year, once per month, once per week, several times per week, or daily. Furthermore, they were asked how much of each type they drank per occasion. The response options were, for beer: no beer, < 1 litre, ≥1 litre; for wine: no wine, <0.5 litre, ≥0.5 to <1 litre, ≥1 litre; for vodka: no vodka, <200 g, ≥200 g (MIHDPS study); or: no vodka, <100 g, ≥100 g (KRIS study).

### Cancer ascertainment and follow-up

Study participants were followed from 1 January 1978 to 31 December 2008. We identified cases of gastric cancer from the Lithuanian Cancer Registry, which has population-based information available since 1978. In addition, deaths from gastric cancer were identified in the National and Regional Archives on Causes of Death. For the present study, the gastric cancer codes were 151.0 to 151.9 of ICD-9 or C16.0 to C16.9 of ICD-10 (International Statistical Classification of Diseases, 9th or 10th Revision respectively). The ascertainment of dates of death and dates of emigration was accomplished by linkage to the Lithuanian Residents’ Register Service.

**Figure 1:**
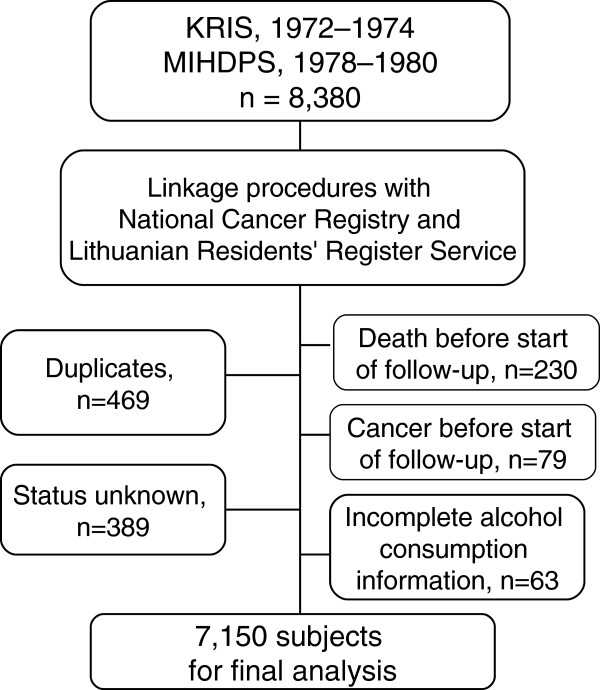
Flow diagram of data management and selection of subjects for the study.

A description of the follow-up is presented in Figure 1. In all, 8,380 cohort members were available for analysis. We excluded 1,230 men because of unknown vital status (4.6%), death before start of follow-up (2.7%), cancer other than nonmelanoma skin cancer before start of follow-up (0.9%), duplicates (5.6%) or incomplete alcohol consumption information (0.8%). Complete data on alcohol consumption was available for 7,150 men.

### Statistical analyses

The association between alcohol consumption and gastric cancer incidence was evaluated using multivariate Cox proportional hazard models. Attained age in months was used as time-scale. Time at entry was the age at the beginning of follow-up, exit time was the age when participants were diagnosed with cancer, died, were lost to follow-up, or were censored at the end of the follow-up period, whichever came first. Study subjects were followed from 1 January 1978 to 31 December 2008. For the MIHDPS study, to reduce the possibility of reverse causation, we excluded the first 3 years of follow-up. The Cox models were stratified by study*,* using ‘study’ as a stratification variable, to control for study-specific effects.

Participants were grouped into four groups on the basis of answers to the question on frequency of alcohol consumption. To improve precision groups were merged into: non-drinkers (i.e. never or former drinkers), a few times per year, 1–4 times per month (i.e. once per month to once per week), 2–7 times per week (i.e. a few times per week to daily). The measures of average weekly intake of ethanol (in grams) from all alcoholic beverages or from specific beverages were calculated on the basis of reported amounts and frequencies of drinking. Alcohol intake categories were converted into the number of alcohol units, and then into grams of ethanol per week, by calculating the dose as the mid-point of each category. For upper open-ended categories, the lower limit was multiplied by 1.2 [20]. A unit was defined as 10 g of ethanol [28]. Individuals were classified into five groups according to their amount of ethanol consumed from all beverages or from specific beverages: non-drinkers, 0.1–9.9 g/week, 10.0–24.9 g/week, 25.0–99.9 g/week and ≥100 g/week. The cut-points were selected on the basis of cohort distribution and aiming to retain extreme categories and sufficient number of cases in sub-groups. For analyses by type of alcoholic beverage, we merged some categories for higher consumption due to limited number of gastric cancer cases. In addition, alcohol intake was converted into milligrams of acetaldehyde per week using acetaldehyde content for each type of alcoholic beverage (beer, wine and vodka) according to Lachenmeier et al. [21]: beer - 18 g/hl of pure alcohol, wine – 28 g/hl of pure alcohol, vodka – 0.7 g/hl of pure alcohol. Weekly acetaldehyde consumption was calculated by combining the frequency of drinking and amount of total acetaldehyde consumption per one occasion. Individuals who consumed alcohol were categorized into approximate quintiles based on the acetaldehyde intake distribution observed in the cohort. The cut-off points used were 0.10, 0.21, 0.86 and 4.11 mg/week. We assumed that occasional drinkers or participants with very light alcohol consumption would have the lowest risk of gastric cancer because some non-drinkers may have quit due to health problems possibly related to gastric cancer risk. Our assumption is based on earlier results from the meta-analysis, where j-shaped dose–response relationship was found and on evidence from EPIC cohort analysis, where non-consumers and former drinkers were at elevated risk for gastric cancer [19,20]. Thus, occasional drinkers (who drank alcohol a few times per year) or participants with very light alcohol consumption (0.1–9.9 g ethanol/week or 0.01–0.10 mg acetaldehyde/week) were used as a reference. Risk estimates for beer, wine and vodka used lighter drinkers of each alcohol type as a reference.

We computed Cox proportional hazard models to obtain Hazard Ratios and 95% Confidence Intervals for alcohol consumption variables, adjusted for potential confounders such as cigarette smoking (never, former, ≤10 cig/day, 11–19 cig/day, ≥20 cig/day), education level (primary, unfinished secondary, secondary and higher), body mass index (BMI) (as a continuous variable). In the analysis of beer, wine and vodka consumption, all beverages were included simultaneously in the model. Because it has been suggested, that the acetaldehyde should be considered as confounding factor in epidemiological studies on alcohol consumption [21], the analysis for ethanol and acetaldehyde intake was repeated with mutual adjustment for these factors. In addition, adjustment for cholesterol and physical activity did not change materially the results, so these variables were not included in the final statistical model. For categorical covariates for which information was missing, we assigned a separate category, while missing data for continuous variable was replaced by the sample mean. Less than 2.5% of the cohort lacked data for each covariate. After excluding non-drinkers, we tested for linear trend by fitting ordinal alcohol consumption variables as continuous. Additionally, standard deviation (SD) scores for the ethanol intake (g/week) and acetaldehyde intake (mg/week) variables were calculated and the multivariable-adjusted HRs associated with an increase in 1 SD were estimated. We also calculated the corresponding HR estimates for gastric cancer for the second half of the follow-up period (1993–2008) to better assess the long-term effect of alcohol consumption. Cancer incidence per 10,000 person-years of follow-up was calculated by dividing the number of cancer cases by the total number of person-years and multiplying by 10,000. To determine the public health impact of heavy drinking, we calculated the population attributable fraction (AF) of heavy alcohol consumption (≥100.0 g/week) on gastric cancer risk using the formula: AF = [P(RR – 1)]/RR, where P is the proportion of cases exposed to a given exposure category of alcohol and RR is the adjusted relative risk for this category [29]. The AF is the estimate of the fraction of cases that would not have occurred if exposure had not occurred [29]. We tested the proportional hazards assumption using the Schoenfeld test. There was no evidence that the proportional hazards assumption was violated for any of the exposure and adjustment variables. We assessed for the interactions between alcohol variables and covariates by using likelihood ratio test (none of the interactions were significant). Analyses were performed using STATA 10. All tests were two-tailed, and statistical significance was assessed at the 5% level.

## Results

Characteristics of the study population according to reported alcohol consumption frequency are shown in Table 1. Men within the group of the highest frequency of alcohol consumption were more likely to smoke and to smoke heavily, and less likely to have a higher education. About 8% of participants were non-drinkers, and 5.6% of participants consumed alcohol a few times per week to daily (daily consumption of alcohol reported 90 (1.2%) individuals). The proportion of men who reported consumption of a particular alcoholic beverage was 16.8% for wine, 89.5% for vodka, and 32.5% for beer. Higher quantity per one occasion reported 16.1% of beer consumers, 16.9% of wine consumers and 74.0% of vodka consumers. There were 185 gastric cancer cases during the follow-up period corresponding to 137,187 person years.

**Table 1:** Selected characteristics of cohort members by alcohol consumption frequency

**Characteristic**	**Alcohol consumption frequency**			
	**Non-drinkers**	**A few times per year**	**1–4 times per month**	**2–7 times per week**
**Cases, n (%)**	11 (5.9%)	27 (14.6%)	132 (71.4%)	15 (8.1%)
**Participants, n (%)**	582 (8.1%)	1239 (17.3%)	4930 (69.0%)	399 (5.6%)
**Person-years**	10,761	24,947	94,719	6,759
**Age at baseline (years)**^a^	53.5±5.6	53.1±5.7	52.3±5.8	53.1±5.7
**Age at diagnosis (years)**^a^	67.6±5.9	65.8±9.2	66.7±8.4	68.5±6.1
**BMI (kg/m**^**2**^**)**^a^	27.4±3.7	27.2±3.6	27.4±3.8	26.8±4.0
**Current smoking (%)**				
Never	40.9	45.1	25.7	16.5
Former	33.8	26.6	22.5	18.5
Smoker:				
≤10 cig/day	9.6	11.7	14.8	12.0
11–19 cig/day	4.3	4.5	9.9	8.8
≥20 cig/day	10.5	10.8	24.9	42.6
Unknown	0.9	1.2	2.2	1.5
**Alcohol intake, g/week**^a^	0	2.5±1.1	56.7±22.1	432.5±195.1
**Married (%)**	93.5	95.8	95.1	93.5
**Education (%)**				
Primary	24.0	15.5	23.5	34.3
Unfinished secondary	27.7	26.3	28.5	29.1
Secondary	26.5	26.7	25.8	26.3
Higher	20.8	30.2	21.1	8.0
Unknown	1.0	1.3	1.1	2.3

^a^Data are presented as mean ± SD.

### Effect of drinking frequency and ethanol amount

Compared to men who drank a few times per year, men in the highest category of alcohol drinking frequency (2–7 times per week) had a statistically significant increased risk of gastric cancer (Table 2). The hazard ratio for developing cancer among men who consumed ≥100.0 g/week of ethanol was a statistically significantly increased compared to men who drank 0.1–9.9 g/week, furthermore, the multivariate adjusted HR per 1 SD (90 g/week) increase in ethanol intake was statistically significant (Table 2). The relationship was strengthened after further adjustment for acetaldehyde intake: for heavy (≥100.0 g/week) ethanol intake HR was 2.82 (95% CI: 1.08–7.41), although, addition of acetaldehyde intake variable to a model did not markedly increase the predictive capacity of the model, p = 0.13 (data not shown). In this cohort, during the 30-year follow-up period, 8.4% of the cancer cases would not have occurred if heavy alcohol consumption (≥100.0 g/week) had not occurred.

**Table 2:** Hazard ratios of gastric cancer incidence according to alcohol consumption

**Alcohol category**	**No. of cases**	**Person-years**	**Incidence rate/10,000 person-years**	**HR (95% CI)**^a^	**HR (95% CI)**^b^
**Follow-up from 1978 through 2008**					
**Frequency**					
Non-drinkers	11	10,761	10.2	0.95 (0.47–1.92)	0.96 (0.47– 1.93)
A few times per year	27	24,948	10.8	1 (reference)	1 (reference)
1–4 times per month	132	94,719	13.9	1.37 (0.90–2.08)	1.27 (0.83–1.94)
2–7 times per week	15	6,759	22.2	2.19 (1.16–4.13)	2.00 (1.04–3.82)
*p-trend*^c^					0.11
**Ethanol intake**					
Non-drinkers	11	10,761	10.2	0.94 (0.47–1.89)	0.95 (0.47–1.90)
0.1–9.9 g/week	29	26,574	10.9	1 (reference)	1 (reference)
10.0–24.9 g/week	56	48,660	11.5	1.10 (0.70–1.72)	1.04 (0.66–1.64)
25.0–99.9 g/week	56	34,858	16.1	1.57 (1.00–2.46)	1.44 (0.91–2.29)
≥100.0 g/week	33	16,334	20.2	2.07 (1.26–3.42)	1.90 (1.13–3.18)
Continuous, per 1 SD (90 g/week)^c^					1.12 (1.00–1.25)
**Follow-up from 1993 through 2008**					
**Frequency**					
Non-drinkers	7	9,676	7.2	1.32 (0.52–3.30)	1.34 (0.53–3.36)
A few times per year	13	23,282	5.6	1 (reference)	1 (reference)
1–4 times per month	79	86,369	9.2	1.88 (1.04–3.39)	1.81 (1.00–3.29)
2–7 times per week	11	5,917	18.6	3.95 (1.76–8.84)	3.99 (1.75–9.11)
*p-trend*^c^					0.007
**Ethanol intake**					
Non-drinkers	7	9,676	7.2	1.20 (0.49–2.95)	1.23 (0.50–3.01)
0.1–9.9 g/week	15	24,707	6.1	1 (reference)	1 (reference)
10.0–24.9 g/week	32	44,576	7.2	1.32 (0.71–2.44)	1.29 (0.69–2.39)
25.0–99.9 g/week	36	31,626	11.4	2.13 (1.16–3.88)	2.06 (1.11–3.81)
≥100.0 g/week	20	14,658	13.6	2.74 (1.40–5.36)	2.74 (1.37–5.49)
Continuous, per 1 SD (90 g/week)^c^					1.17 (1.03–1.32)

^a^Stratified by study. Age was used as the underlying time metric for all analyses.

^b^Stratified by study and adjusted for smoking (never, former, ≤10 cig/day, 11–19 cig/day, ≥20 cig/day); education (primary, unfinished secondary, secondary, higher); BMI. Age was used as the underlying time metric for all analyses.

^c^Test for trend was carried out after exclusion of non-drinkers.

A total of 110 gastric cancer cases were identified during the second half of the follow-up period from January 1993 through December 2008 among the 5,425 men who were alive on 1 January 1993. Similar to the results based on the whole cohort, alcohol consumption was significantly associated with an increased risk of gastric cancer; furthermore, the association became stronger (Table 2).

In the multivariate analysis, smoking was associated with a non-significantly increased risk of gastric cancer, HR = 1.48 (95% CI: 0.88–2.49) for men who smoked 10–19 cigarettes per day and 1.39 (95% CI: 0.92–2.10) for men who smoked ≥20 cigarettes per day (compared to never smokers).

### Type of alcoholic beverage

**Figure 2:**
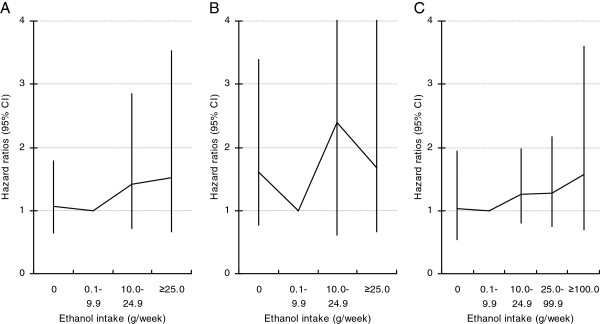
**Hazard ratios (95% CI) of gastric cancer by ethanol intake for each alcoholic beverage type: beer (A), wine (B) and vodka (C).** Models stratified by study; adjusted for smoking, education, BMI. Mutually adjusted for the other two beverage types.

Higher consumption of beer or vodka per one occasion was not statistically significantly associated with a risk of gastric cancer in this cohort of men (Table 3). The gastric cancer incidence rate for heavy wine drinkers was very high in comparison with heavy beer drinkers or heavy vodka drinkers (Table 3). In a multivariate adjusted model, heavy wine consumption was positively related to a risk of gastric cancer, HR = 2.95 (95% CI: 1.30–6.68) for men who consumed ≥0.5 litre per occasion compared with those who consumed less (based on 10 cases, mutually adjusted for beer and vodka) (Table 3). In an analysis by ethanol intake from each type of alcoholic beverage, after adjustment for smoking, education level, BMI and the other two beverage types, HRs for the highest category of beer, wine and vodka consumption were not significantly elevated, HR = 1.52 (95% CI: 0.66–3.51), based on 16 cases, 1.72 (95% CI: 0.67–4.40), based on 17 cases, and 1.50 (95% CI: 0.66–3.42), based on 13 cases, respectively (Figure 2). Tests for trend were statistically not significant.

**Table 3:** Hazard ratios of gastric cancer by type of alcoholic beverage and intake per one occasion

**Alcoholic beverage**	**No. of cases**	**Person-years at risk**	**Incidence rate/10,000 person-years**	**HR (95% CI)**^a^	**HR (95% CI)**^b^
**Beer**					
0	121	93,036	13.0	0.84 (0.59–1.20)	0.83 (0.58–1.18)
<1 litre	54	37,541	14.4	1	1 (reference)
≥1 litre	10	6,611	15.1	0.82 (0.40–1.67)	0.79 (0.39–1.62)
*p-trend*^c^					0.60
**Wine**					
0	156	115,296	13.5	1.50 (0.89–2.53)	1.47 (0.87–2.50)
<0.5 litre	19	18,636	10.2	1	1 (reference)
≥0.5 litre	10	3,254	30.7	3.18 (1.41–7.14)	2.95 (1.30–6.68)
*p-trend*^c^					0.65
**Vodka, MIHDPS**^**d**^					
0	14	11,298	12.4	1.22 (0.65–2.30)	1.20 (0.63–2.25)
<200 g	31	29,633	10.5	1	1 (reference)
≥200 g	97	67,291	14.4	1.42 (0.94–2.13)	1.25 (0.82–1.92)
*p-trend*^c^					0.55
**Vodka, KRIS**					
0	1	2,466	4.0	0.37 (0.04–3.29)	0.37 (0.04–3.36)
<100 g	4	3,929	10.2	1	1 (reference)
≥100 g	38	22,570	16.8	1.69 (0.60–4.79)	1.51 (0.52–4.36)
*p-trend*^c^					0.11

^a^Stratified by study. Mutually adjusted for the other two beverage types.

^b^Stratified by study and adjusted for smoking (never, former, ≤10 cig/day, 11–19 cig/day, ≥20 cig/day); education (primary, unfinished secondary, secondary, higher); BMI. Mutually adjusted for the other two beverage types. Age was used as the underlying time metric for all analyses.

^c^Test for trend was carried out across three categories by fitting ordinal variable ‘amount per one occasion’ as continuous.

^d^Results for MIHDPS and KRIS participants are presented separately due to differences in questionnaire design.

### Acetaldehyde intake

**Figure 3:**
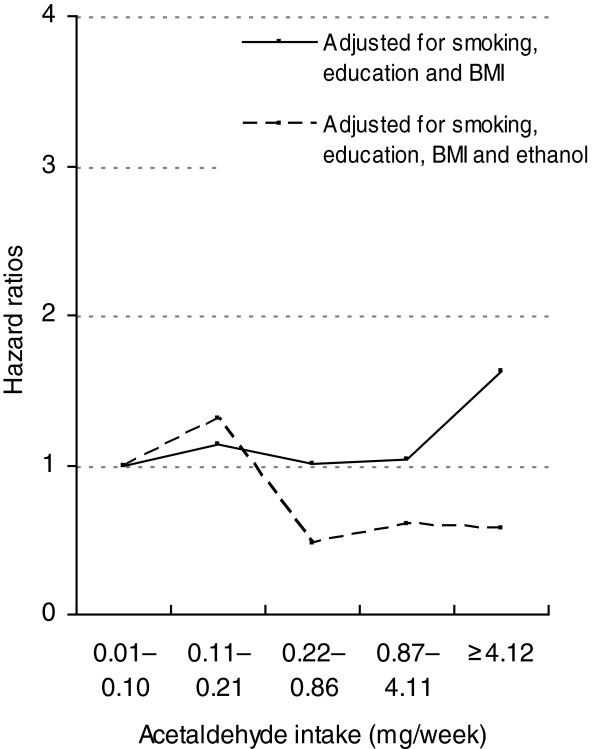
**Hazard ratios of gastric cancer by acetaldehyde intake among alcohol consumers.** Models stratified by study; adjusted for smoking, education, BMI, before and after adjustment for ethanol intake.

Figure 3 shows the association between total acetaldehyde intake and the gastric cancer in the multivariate analyses. HR adjusted for smoking, education level and body mass index among men within the 5th quintile was significantly increased compared to those within the 1st quintile (HR = 1.66 (95% CI: 1.04–2.65). The HR per 1 SD (11.8 mg of acetaldehyde per week) was 1.08 (95% CI: 0.98–1.19). The association for intake of acetaldehyde did not persist after inclusion of ethanol intake in the model (HR = 0.60 (95% CI: 0.24–1.52), trend analysis showed no dose–response relationship; p value for the addition of the ethanol intake variable was <0.05.

## Discussion

In this population-based cohort study with a long follow-up period, a significant association between alcohol consumption and increased risk of gastric cancer was observed. Men within the group of the highest alcohol consumption frequency had a twofold increased risk of gastric cancer compared with occasional drinkers. A statistically significant positive association between the amount of ethanol intake and gastric cancer was also observed. Our results suggest that the fraction of cancer cases attributable to alcohol consumption in this cohort is approximately 8%.

Previous studies have reported inconsistent results [5,6,8-20,30-32]. A positive association with heavy alcohol drinking was observed in a prospective study of men from China (HR = 1.46 (95% CI: 1.05–2.04)) and in men from a European Prospective Investigation into Cancer and Nutrition (EPIC) study (HR = 1.65 (95% CI: 1.06–2.58)) [18,19]. Furthermore, a meta-analysis of 44 case–control and 15 cohort studies showed a relationship with heavy drinking, HR = 1.20 (95% CI: 1.01–1.44) [20]. Our results showed that higher alcohol consumption increases gastric cancer risk. This observation is in agreement with those studies that found an association between alcohol drinking and the risk of gastric cancer [14,16-20].

A stronger effect of alcohol consumption on gastric cancer risk was observed in our study during the period 1993–2008. We cannot rule out the possibility that an increasing alcohol intake over time among study participants could influence these results. According to a WHO MONICA study carried out in Kaunas among men 35–64 years of age, the frequency of alcohol consumption and average amount drunk increased markedly in 2001–2002 compared to 1983–1984, despite some reduction noticed in 1986–1993 [33]. Another possible explanation for this is that a longer latency period was associated with a stronger relationship between alcohol consumption and risk of gastric cancer.

Our study observed an increased risk among greater consumers of wine. Men who consumed ≥0.5 litre of wine per occasion had an increased risk of gastric cancer compared with those who consumed less. In addition, a very high incidence rate was observed among heavy wine consumers but not among heavy drinkers of the other beverage types, supporting the association between the heavy consumption of wine and gastric cancer. When we estimated wine consumption using a measure that combines quantity and frequency (i.e. grams of ethanol per week), we observed increased risk, but not statistically significant. Thus, our data suggest the greater importance of quantity of wine consumption. However, we cannot rule out the possibility that the significant risk increase among greater consumers of wine was a chance finding due to relatively small number of cases in wine drinkers. In analyses of the effect of estimated ethanol intake by beverage type, we found no significant association with beer or vodka consumption. There are mixed findings about the relation between the type of alcoholic beverage and the incidence of gastric cancer. Wine drinking was significantly associated with a risk of developing gastric cancer in a study in Mexico, for the highest category of wine intake Odds ratio (OR) = 2.93 (CI 95%: 1.27–6.75) [34]. In Portugal, consumption of more than one bottle of red wine per day 20 years prior to the interview was also associated with a gastric cancer risk, OR = 2.61 (p = 0.049) [35]. In Lithuania, a case–control study demonstrated a positive association between wine consumption and gastric cancer risk, but no association with beer or spirits [36]. In contrast, several studies showed that wine may be comparatively less carcinogenic, and a daily intake of wine may prevent the development of gastric cancer [11,37]. No association with wine or liquor and gastric cancer risk was found in a recent EPIC cohort, but there was a positive association for beer, HR = 1.75 (95% CI: 1.13– 2.73) for ≥30 g/day [19]. A strong effect of alcohol, particularly vodka consumption, was observed in case–control studies of gastric cancer in Russia and Uruguay [16,17]. No association with alcohol intake was found in a study from Poland, although it was suggested that heavy drinkers with a slow elimination of acetaldehyde due to polymorphism in alcohol metabolizing gene (ALDH2) had an excess risk [6,38].

Several mechanisms have been proposed to explain the possible role of alcohol in gastric cancer development. The ethanol in alcoholic beverages is considered to be “the principal ingredient that renders these beverages carcinogenic” [4,21]. Ethanol induces various reactive oxygen species and oxidative stress, which damage the DNA and affect its repair. Chronic ethanol intake is known to induce cytochrome P450 2E1 (CYP2E1) in various organs, including gastrointestinal tract, which affect conversion of pro-carcinogens (present in alcoholic beverages, tobacco smoke and diet) into carcinogens [4,39,40]. Ethanol may further act as a solvent for these carcinogens to enter the cell in the mucosa of the stomach, it may also have direct physical effect on the tissue [39]. In the body, ethanol is converted by alcohol dehydrogenase and CYP2E1 to acetaldehyde. Acetaldehyde may promote carcinogenesis by causing point mutations, inducing sister-chromatid exchanges, impairing DNA repair, inducing metaplasia of epithelium, and forming mutagenic adducts with DNA [40]. It has been shown, that acetaldehyde outside ethanol metabolism poses a risk for drinkers of alcoholic beverages above the risk of ethanol and metabolically formed acetaldehyde [21]. In addition, beer consumption results in exposure to volatile N-nitroso compound N-nitrosodimethylamine (NDMA), which is a potent carcinogen in animals [41].

One hypothesis that might explain the high risk of gastric cancer associated with alcohol in Lithuania is the presence of contaminants, e.g. acetaldehyde, which has been classified as carcinogenic to humans (Group 1) [4,21,42]. There is no data on the NDMA levels in beer on the market in Lithuania; however, high levels of acetaldehyde have been detected in fruit-based liquor samples from Central European countries [43]. Epidemiological evidence on acetaldehyde as an independent risk factor for cancer during alcohol consumption, in addition to the effects of ethanol, is limited. We addressed the possibility that acetaldehyde consumption may confer some increased risk of gastric cancer or may be a confounding factor. This has, to our knowledge, not been previously examined. We repeated analyses of ethanol intake adjusting for total acetaldehyde intake. Furthermore, we performed analysis of acetaldehyde with and without adjustment for ethanol intake. In those analyses results suggested increased risk with ethanol intake and no association with acetaldehyde intake after mutual adjustment for these factors. It is noteworthy, that non-alcoholic components of beverages could not be considered in the present analysis as confounding factors. Wine has the highest acetaldehyde content, but it also contains substances thought to be protective. Therefore, it is likely that when both acetaldehyde and ethanol were included in the same statistical model as covariates, reduced HRs for acetaldehyde were obtained due to this confounding. In contrast, the analysis by beverage type revealed increased gastric cancer risk among higher consumers (≥0.5 litre per one occasion) of wine suggesting that the relation could be attributed to the higher content of acetaldehyde in wine. Thus, interpretation is difficult given positive correlation between ethanol and acetaldehyde intake and limited statistical power because the majority of the cohort participants reported no or low consumption of wine or beer. Since wine and beer have higher acetaldehyde concentrations than do white spirits (vodka) [21], and since many countries have higher consumption of wine and beer, this does not rule out an acetaldehyde effect among more heavily exposed alcohol consumers.

Here we only studied men and it may be, that these results are not directly applicable to women. Previous studies reported conflicting results on alcohol consumption and gastric cancer risk by gender: two studies revealed higher risk associated with alcohol consumption among men than among women [11,16], in one an association was significant only in men but not in women [19], whereas one found no significant differences in risk among men and women [20].

The major strengths of this study include its prospective and population-based design, long follow-up time, and the completeness of follow-up through the use of population-based registers. In addition, we controlled for a range of potential confounding variables, including smoking, body mass index, education, age and acetaldehyde intake. The study also has limitations. The first limitation is the lack of data on diet, alcohol use in the past, Helicobacter pylori infection status, and histological subtype or cancer anatomical subsite. Fresh fruit and vegetable intake is inversely, whereas red and processed meat and salt intake is positively, associated with the risk of gastric cancer [2]. An increased risk associated with heavier drinking might be due to poor nutrition, as heavy drinkers are known to have less healthy dietary habits [44]. We cannot rule out the possibility that the observed associations between alcohol consumption and gastric cancer risk were confounded by factors that we have not accounted for. However, in several other studies, the adjustment for potentially confounding variables, including dietary factors or *H. pylori* serology, did not materially change the association between alcohol consumption and risk of gastric cancer [12,18,20]. The second limitation is the assessment of alcohol intake at a single point in time. There is a potential for misclassification of exposure due to possible changes in alcohol intake over time. If alcohol consumption increased with time, this could lead to an overestimation of the risk among moderate drinkers. The third limitation is the assessment of alcohol intake based on a questionnaire, therefore underreporting or misclassification might have occurred. Our ability to assess relationships was limited by the lack of detailed questionnaire on alcohol consumption. Furthermore, we had no information on specific type of spirits, thus we were unable to take into account an acetaldehyde intake from fruit-based spirits. However, findings indicate that the traditional vodka drinking culture prevaled at the time of baseline surveys [45]. Because of the prospective design of the present study, any misclassification of alcohol intake would be non-differential and would tend to dilute the true association. The further limitation is that the results of analyses were based on a small number of gastric cancer cases. Thus, it cannot be excluded that the associations were observed by chance.

## Conclusions

The results from the present cohort study support an association between higher consumption frequency or higher alcohol intake and the risk of gastric cancer among men. This prospective study suggests that the effect was due to total ethanol intake. Although an excess risk among men within the highest wine consumption group was observed, an association between exposure to acetaldehyde and risk of gastric cancer in this cohort was not confirmed. Our findings imply that public health recommendations to reduce the gastric cancer burden need to include a reduction in alcohol consumption. However, further research is needed to provide a more detailed evaluation of alcohol drinking, possible important confounding factors and anatomical subsites of gastric cancer.

## Competing interests

The authors declare that they have no competing interests.

## Authors’ contributions

The study was designed and coordinated by RE, AT and RRe. Data acquisition and cleaning was performed by all the authors. DV, RRa and EM prepared the final database. DV, RE, IK, AT analyzed and interpreted the data. The manuscript was prepared by RE, AT, IK. All authors read and approved the final manuscript.

## Pre-publication history

The pre-publication history for this paper can be accessed here:

http://www.biomedcentral.com/1471-2407/12/475/prepub
